# Chromatin remodeling protein MORC2 promotes breast cancer invasion and metastasis through a PRD domain-mediated interaction with CTNND1

**DOI:** 10.18632/oncotarget.18556

**Published:** 2017-06-16

**Authors:** Xiao-Hong Liao, Ye Zhang, Wen-Jie Dong, Zhi-Min Shao, Da-Qiang Li

**Affiliations:** ^1^ Shanghai Cancer Center and Institutes of Biomedical Sciences, Shanghai Medical College, Fudan University, Shanghai 200032, China; ^2^ Department of Oncology, Shanghai Cancer Center, Shanghai Medical College, Fudan University, Shanghai 200032, China; ^3^ Cancer Institute, Shanghai Cancer Center, Shanghai Medical College, Fudan University, Shanghai 200032, China; ^4^ Department of Breast Surgery, Shanghai Cancer Center, Shanghai Medical College, Fudan University, Shanghai 200032, China; ^5^ Key Laboratory of Breast Cancer in Shanghai, Shanghai Medical College, Fudan University, Shanghai 200032, China

**Keywords:** breast cancer, invasion and metastasis, protein-protein interaction, MORC2, proline-rich domain

## Abstract

MORC family CW-type zinc finger 2 (MORC2) is a newly identified chromatin remodeling protein with emerging roles in the regulation of DNA damage response and gene transcription, but its mechanistic role in breast cancer development and progression remains unexplored. Here, we show that MORC2 promoted breast cancer invasion and metastasis and these effects depended on a proline-rich domain (PRD) within its carboxy-terminal region spanning residues 601–734. Induced expression of wild-type MORC2 did not significantly affect cell proliferation and cell-cycle progression, but promoted breast cancer cell migration and invasion *in vitro* and metastatic lung colonization *in vivo*. The PRD domain was dispensable for the protein stability and subcellular localization of MORC2, but depletion of the PRD domain substantially suppressed MORC2-mediated migration, invasion, and metastasis. Proteomic and biochemical analyses further demonstrated that wild-type MORC2, but not PRD deletion mutant, interacted with catenin delta 1 (CTNND1), a cadherin-associated protein that participates in tumor invasion and metastasis. Moreover, knockdown of endogenous CTNND1 by short hairpin RNAs suppressed the migratory and invasive potential of MORC2-expressing cells. Taken together, these results suggest that MORC2 promotes breast cancer invasion and metastasis through its PRD domain-mediated interaction with CTNND1.

## INTRODUCTION

Breast cancer is the most commonly diagnosed cancer and the leading cause of cancer mortality in women worldwide [1]. Clinical evidence shows that over 90% of breast cancer-related death is attributable to distant metastasis to target organs including the lungs [2], liver [3], bones [4], or brain [5]. Despite the significant clinical challenge, the molecular mechanism underlying breast cancer invasion and metastasis has not yet been fully delineated.

MORC family CW-type zinc finger protein 2 (MORC2) is a member of the microrchidia (MORC) nuclear protein superfamily [6]. Although MORC2 is ubiquitously expressed in human cells and tissues [7–9], its biological functions in mammalian cells remain largely unknown. Recently, we and others defined MORC2 as a global chromatin remodeler with emerging roles in the regulation of DNA damage response [7] and gene transcription [8, 10, 11]. In addition to its nuclear functions, cytosolic MORC2 is implicated in the regulation of lipogenesis and adipocyte differentiation [12]. In human cancer, MORC2 has been shown to promote gastric tumorigenesis [10, 11, 13]. Interestingly, two recent gene expression profiling studies revealed that the expression levels of MORC2 are up-regulated in breast cancer tissues as compared with adjacent normal breast tissues [14] and are associated with recurrence risk of patients with highly aggressive triple-negative breast cancer [15]. However, the mechanistic role for MORC2 in breast cancer development and progression remains unexplored.

The proline-rich domain (PRD) was first identified in the p53 tumor suppressor protein as a region enriched in prolines and containing several repeats of the amino acid motif PXXP (where P indicates proline and X indicates any amino acid) [16]. Subsequent studies further demonstrated that the PRD domain is widely distributed in eukaryotic proteomes and is usually involved in the assembly of multi-protein complexes [17–19]. Moreover, emerging evidence shows that the PRD domain is intimately implicated in the spatial and temporal control of diverse signal transduction events through interaction with specific binding modules, such as the Src homology 3 (SH3), WW, and Enabled/VASP homology-1 (EVH1) domains [20]. Interestingly, analysis of the MORC2 protein sequence revealed the presence of a putative PRD domain within its carboxy-terminal region spanning residues 601–734, but the biological functions of the PRD domain in MORC2 remain largely unknown.

Catenin delta 1 (CTNND1), also known as p120-catenin, was originally identified as a substrate of the oncogenic tyrosine kinase Src [21] and subsequently defined as a component of the adherens junction complex that includes E-cadherin and α-, β-, γ-catenins [22, 23]. Of interest, CTNND1 is a multifaceted intracellular signaling protein, which may function as either a tumor suppressor or a metastasis promoter depending on its subcellular localization and E-cadherin expression status [24, 25]. In this context, a core function of CTNND1 in mammalian cells is to stabilize E-cadherin at cell membrane by preventing its endocytosis and degradation in the lysosome [26–28], thus facilitating E-cadherin-mediated suppression of tumor invasion and metastasis [27, 29–31]. In turn, E-cadherin is both necessary and sufficient for localization of CTNND1 at cell membrane [32, 33]. Consequently, loss of E-cadherin function or expression during tumor progression as a consequence of the epithelial to mesenchymal transition results in the translocation of CTNND1 from cell membrane to the cytoplasm and/or the nucleus [33–37]. In the cytoplasm, CTNND1 promotes cell migration and, consequently, tumor invasion and metastasis through activation of Rho-family GTPases Rac1 and Cdc42 and inhibition of RhoA activity [29, 38–43]. Consistently, the increased cytoplasmic localization of CTNND1 is closely associated with the increased invasive phenotype of E-cadherin-deficient breast cancer cells [29, 33, 35, 44]. In addition, CTNND1 protein contains two putative nuclear localization signals and a nuclear export signal, thus outlining a potential role for CTNND1 in nuclear signaling [36, 45, 46]. Indeed, elevated levels of CTNND1 in the nucleus have been observed in E-cadherin-deficient cancer cells [36, 37], indicating a potential role for nuclear CTNND1 in enhancing the metastatic phenotype associated with E-cadherin downregulation [37]. In addition, CTNND1 has been documented to interact with the transcriptional factor Kaiso [46, 47] and regulate the expression of Kaiso target genes involved in canonical and noncanonical Wnt signaling [48–50]. Together, although CTNND1 is recognized as a key regulator in cancer progression and metastasis, the mechanistic aspects of its prometastatic signaling in breast cancer are not fully characterized.

In this study, we addressed the functional and mechanistic roles for MORC2 in breast cancer development and progression. Findings presented here show that MORC2 promotes breast cancer invasion and metastasis, and the PRD domain is essential for the metastasis-promoting properties of MORC2 through interacting with CTNND1.

## RESULTS

### The PRD domain is dispensable for the stability and subcellular localization of MORC2

**Figure 1:**
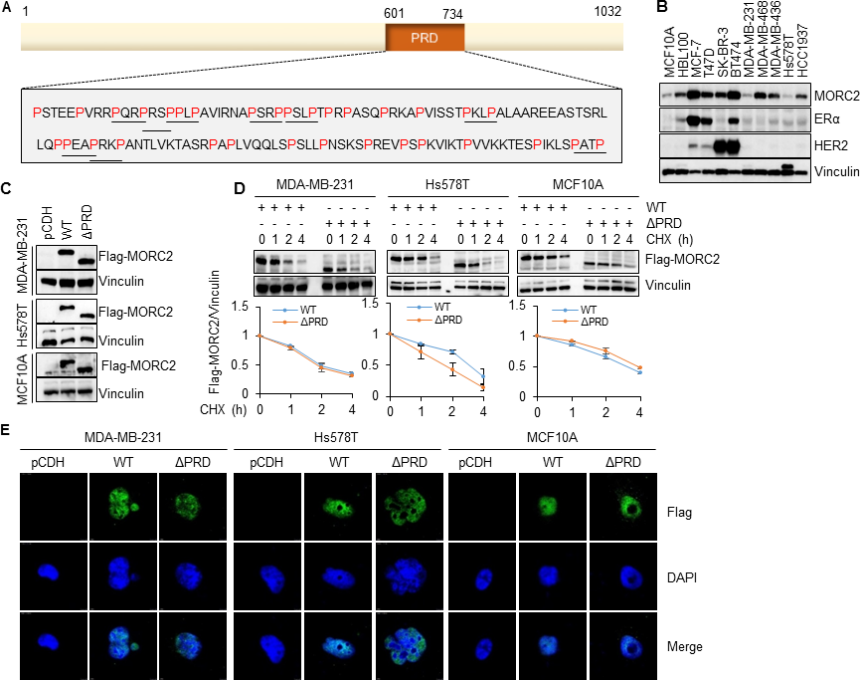
The PRD domain is dispensable for the stability and subcellular localization of MORC2 (**A**) Schematic representation of the PRD domain of human MORC2 protein. P indicates proline. (**B**) Immunoblotting analysis of the expression levels of endogenous MORC2, ERα, and HER2 in 2 normal breast epithelial cell lines and 9 breast cancer cell lines. Vinculin was used as a loading control. (**C**) Establishment of stable MCF10A, MDA-MB-231 and Hs578T cell lines expressing empty vector pCDH, wild-type MORC2 (Flag-MORC2 WT), and PRD deletion mutant MORC2 (Flag-MORC2 ΔPRD) by lentivirus infection. The expression levels of MORC2 were verified by immunoblotting. (**D**) Cells stably expressing Flag-MORC2 WT and Flag-MORC2 ΔPRD were treated with 100 μg/ml of cycloheximide (CHX) for the indicated time points and subjected to immunoblotting analysis with the indicated antibodies. (**E**) Cells were infected with lentiviral vectors encoding pCDH, Flag-MORC2 WT, and Flag-MORC2 ΔPRD for 48 h and then subjected to indirect immunofluorescence staining with an anti-Flag antibody. Cell nuclei were counterstained with DAPI.

Analysis of the MORC2 amino acid sequence revealed the presence of a putative PRD domain within the C-terminal region spanning residues 601–734 (Figure 1A). This region is rich in the amino acid proline (32/134 residues) and contains 9 copies of canonical PXXP motifs (P designating proline and X designating any amino acid) (Figure 1A). As the functional importance of the PRD domain has been documented in multiple cancer relevant proteins such as p53 (Supplementary Figure 1) [16, 19, 51–56], we set out to address the possible functions of the PRD domain in MORC2.

To do this, we first examined the protein expression levels of endogenous MORC2 in normal mammary epithelial cell lines MCF10A and HBL100, and breast cancer cell lines MCF-7, T47D, SK-BR-3, BT474, MDA-MB-231, MDA-MB-468, Hs578T, and HCC1937. As shown in Figure 1B, MCF-7 and BT474 cells showed high levels of endogenous MORC2, whereas MCF10A, MDA-MB-231, and Hs578T cells expressed low levels of endogenous MORC2. Consistent with the literature [57], estrogen receptor α (ERα) was detected in MCF-7, T47D, and BT474 cells, while human epidermal growth factor receptor 2 (HER2) was highly expressed in SK-BR-3 and BT474 cells (Figure 1B), indicating that the cell lines used in this study were well authenticated. Second, we generated a MORC2 cDNA deletion mutant (ΔPRD), which lacks this entire PRD domain (deleted for amino acids 601–734). Third, we chose MCF10A, MDA-MB-231, and Hs578T cell lines, which express low levels of endogenous MORC2 (Figure 1B), to generate stable cell lines expressing empty vector pCDH, wild-type MORC2 (Flag-MORC2 WT), and PRD domain deletion mutant MORC2 (Flag-MORC2 ΔPRD) by lentiviral infection. The expression levels of Flag-MORC2 in the resultant cell lines were verified by immunoblotting (Figure 1C).

Given that the PRD domain has been shown to be involved in the regulation of the subcellular localization and stability of some PRD domain-containing proteins [58, 59], we next examined whether the PRD domain of MORC2 could affect its protein stability and subcellular localization. For this purpose, MCF10A, MDA-MB-231 and Hs578T cells stably expressing Flag-MORC2 WT and Flag-MORC2 ΔPRD were treated with 100 μg/ml cycloheximide (CHX), a protein synthesis inhibitor, for the indicated time points and then subjected to immunoblotting analysis with an anti-Flag antibody. Results showed that there were no significant differences in the expression levels of Flag-MORC2 WT and Flag-MORC2 ΔPRD following CHX treatment at various time points (Figure 1D). To determine whether the PRD domain could affect the subcellular localization of MORC2, MDA-MB-231, Hs578T, and MCF10A cells were transiently infected with the lentiviral expression vectors encoding pCDH, Flag-MORC2 WT, and Flag-MORC2 ΔPRD for 48 h, and then indirect immunofluorescence staining was performed using an anti-Flag antibody. 4′,6-diamidino-2-phenylindole (DAPI) was used to detect the nuclei in all cells. The infection efficiency of lentiviral vectors was about 20%, and about 85% transiently expressed Flag-MORC2 and Flag-MORC2 ΔPRD were localized in the nuclear (Figure 1E and Supplementary Figure 2). Taken together, these results suggest that the PRD domain is dispensable for the stability and subcellular localization of MORC2.

### Both wild-type MORC2 and PRD deletion mutant MORC2 are not required for cell proliferation and cell-cycle progression of breast cancer cells

**Figure 2:**
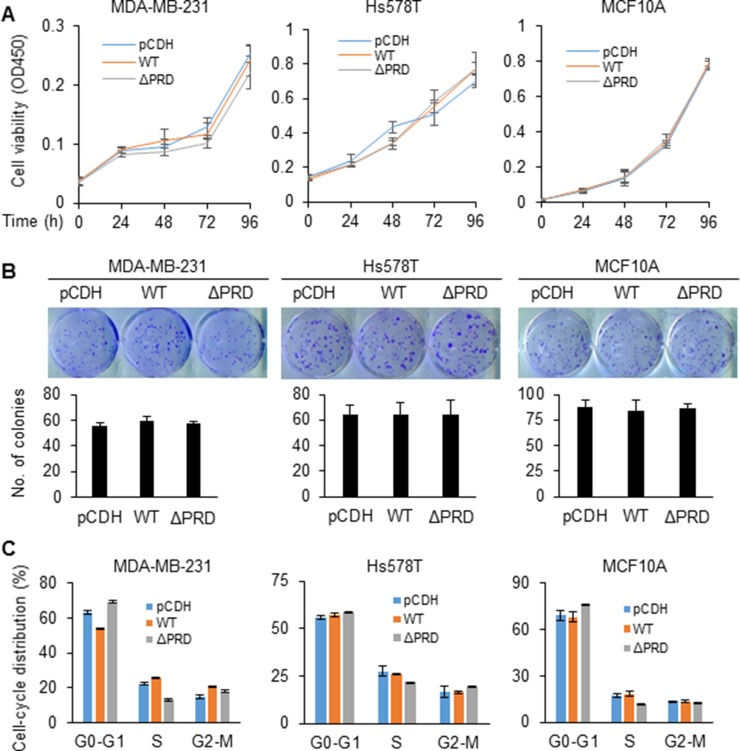
MORC2 and its PRD domain are not required for cell proliferation and cell-cycle progression (**A**) Cell viability was determined at the indicate time points using CCK-8 kit. (**B**) Cells were seeded in 6-well plates and cultured for 2 weeks. Cells were staining with 0.1% crystal violet dye and the numbers of colonies were counted. (**C**) Cell-cycle distribution was analyzed using flow cytometry.

To characterize the function of MORC2 and its PRD domain in breast cancer cells, cell proliferation and cell-cycle progression of MCF10A, MDA-MB-231, and Hs578T cells stably expressing pCDH, Flag-MORC2 WT, and Flag-MORC2 ΔPRD were evaluated using Cell Counting Kit-8 (CCK-8), colony formation assay, and flow cytometry, respectively. The CCK-8 assays revealed that induced expression of either wild-type MORC2 or PRD deletion mutant did not significantly affect cell viability as compared with empty vector pCDH-infected cells (Figure 2A). Consistently, colony formation assays also demonstrated that there were no significant differences in both the size and the numbers of the colonies among cells stably expressing pCDH, Flag-MORC2 WT, and Flag-MORC2 ΔPRD (Figure 2B). In addition, flow cytometry analysis showed that cells stably expressing pCDH, Flag-MORC2 WT, and Flag- MORC2 ΔPRD did not show significant differences in cell-cycle distribution (Figure 2C). Collectively, these results suggest that induced expression of both wild-type MORC2 and PRD deletion mutant MORC2 did not significantly affect cell proliferation, colony formation, and cell-cycle progression.

### Induced expression of wild-type MORC2, not PRD deletion mutant MORC2, promotes breast cancer cell migration, invasion, and metastasis

**Figure 3:**
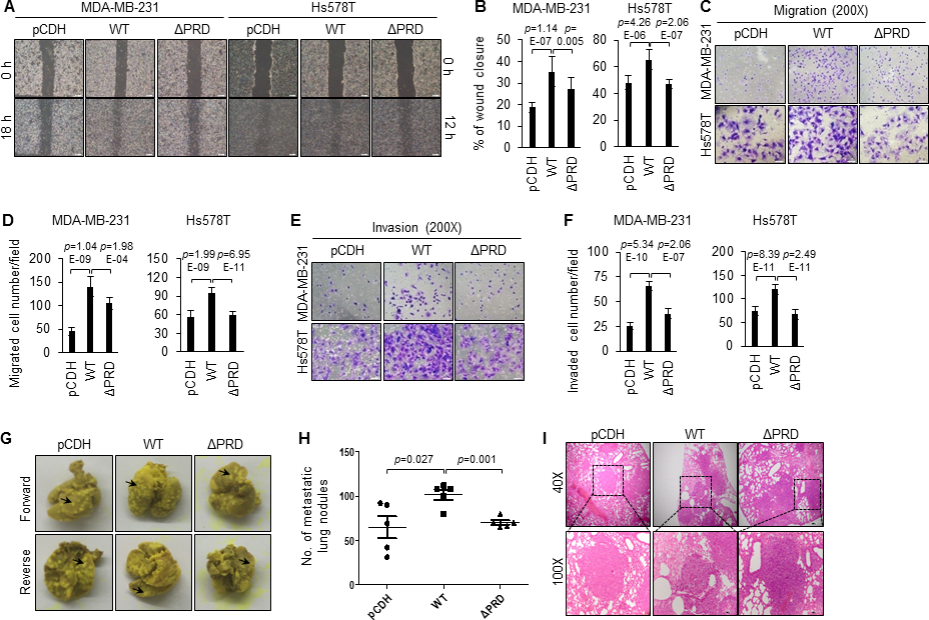
Induced expression of wild-type MORC2, but not PRD deletion mutant MORC2, enhances breast cancer cell migration, invasion and metastasis (**A**, **B**) MDA-MB-231 and Hs578T cells stably expressing pCDH, Flag-MORC2 WT, and Flag-MORC2 ΔPRD were subjected to wound-healing assays. Representative images (A) and quantitative results (B) are shown. (**C**–**F**) MDA-MB-231 and Hs578T cells stably expressing pCDH, Flag-MORC2 WT, and Flag-MORC2 ΔPRD were subjected to transwell migration (C–D) and invasion (E–F) assays. Representative images of cell migration and invasion (C, E) and the corresponding quantitative results (D, F) are shown. (**G**–**I**) MDA-MB-231 cells stably expressing pCDH, Flag-MORC2 WT, and Flag-MORC2 ΔPRD were injected into 5–6 week-old BALB/c female nude mice (5 mice per group) through the tail vein, and lungs were harvested after 6 weeks of injection. Representative images of lung metastasis (G), corresponding quantitative results of lung nodules (H), and representative images of H&E-stained sections of lung tissues (I) are shown.

As one of the hallmarks of breast cancer is its ability to invade and metastasize [2–5], we next sought to determine the impact of MORC2 protein and its PRD domain on the invasive and metastatic capacity of breast cancer cells. To investigate the involvement of MORC2 and its PRD domain in breast cancer cell migration and invasion *in vitro*, wound healing (Figure 3A, 3B), transwell migration (Figure 3C, 3D) and invasion assays (Figure 3E, 3F) were performed. Results showed that MDA-MB-231 and Hs578T cells expressing wild-type MORC2 exhibited significantly increased migration (Figure 3A–3D) and invasion (Figure 3E, 3F) capabilities as compared with pCDH expressing cells. Interestingly, depletion of the PRD domain significantly suppressed breast cancer cell migration and invasion (Figure 3A–3F). These results collectively indicate that MORC2 promotes breast cancer migration and invasion *in vitro* through, at least in part, its PRD domain.

Cell migration and invasion are essential for metastatic dissemination of breast cancer. To test whether MORC2 and its PRD domain affect the ability of breast cancer cells to colonize the lung, MDA-MB-231 cells stably expressing pCDH, Flag-MORC2 WT, and Flag-MORC2 ΔPRD were injected into the tail vein of nude mice and the lung metastasis nudes were examined after 6 weeks of injection. Consistent with *in vitro* experimental findings, induced expression of wild-type MORC2 increased the number of the metastatic lung lesions compared to the empty vector pCDH control (Figure 3G, 3H). In contrast, expression of PRD domain deletion mutant MORC2 reduced the lung metastatic burden (Figure 3G, 3H). These results were further confirmed by analysis of hematoxylin-eosin-stained lung sections (Figure 3I). Together, these data suggests that the PRD domain is important for metastasis-promoting activity of MORC2 *in vivo*.

### MORC2 interacts with CTNND1 in a PRD domain-dependent manner

**Figure 4:**
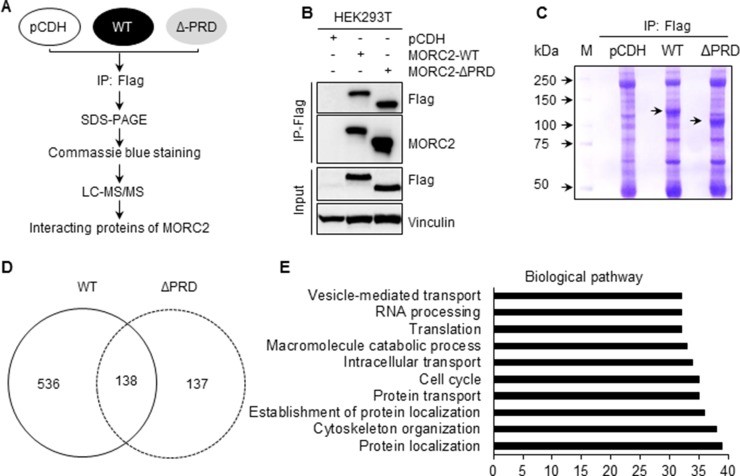
Identification of the binding partners of MORC2 using IP coupled with LC-MS/MS (**A**) Schematic representation of experimental design. (**B**, **C**) HEK293T cells stably expressing pCDH, Flag-MORC2 WT, and Flag-MORC2 ΔPRD were subjected to IP analysis with an anti-Flag antibody (B), and the bound proteins were isolated on 8% SDS-PAGE gel and stained using Coomassie brilliant blue (C). (**D**) LC-MS/MS was used to identify the interacting proteins of Flag-MORC2 WT and Flag-MORC2 ΔPRD. The numbers of the identified proteins in each group are shown. (**E**) The proteins that specifically interacted with Flag-MORC2 WT were subjected to biological pathway analyses using DAVID.

We next investigated the molecular mechanism by which MORC2 promotes breast cancer invasion and metastasis. As the PRD domain is a putative protein-binding module [20], we next identified the potential binding partners of MORC2 by immunoprecipitation (IP) coupled with liquid chromatography tandem mass spectrometry (LC-MS/MS) method (Figure 4A). To do this, HEK293T cells stably expressing pCDH, Flag-MORC2 WT, and Flag-MORC2 ΔPRD were subjected to IP analysis with an anti-Flag antibody (Figure 4B), and the immunoprecipitated proteins were isolated by SDS-PAGE and then stained by Coomassie brilliant blue (Figure 4C). LC-MS/MS analysis showed that 536 proteins specifically interacted with wild-type MORC2, whereas 137 proteins interacted with the PRD deletion mutant MORC2 (Figure 4D). Bioinformatic analysis using DAVID (https://david.ncifcrf.gov) revealed that the biological pathway involving those 536 proteins that specially bind to wild-type MORC2 (Supplementary Table 1) is mainly associated with protein localization and cytoskeleton regulation (Figure 4E).

**Figure 5:**
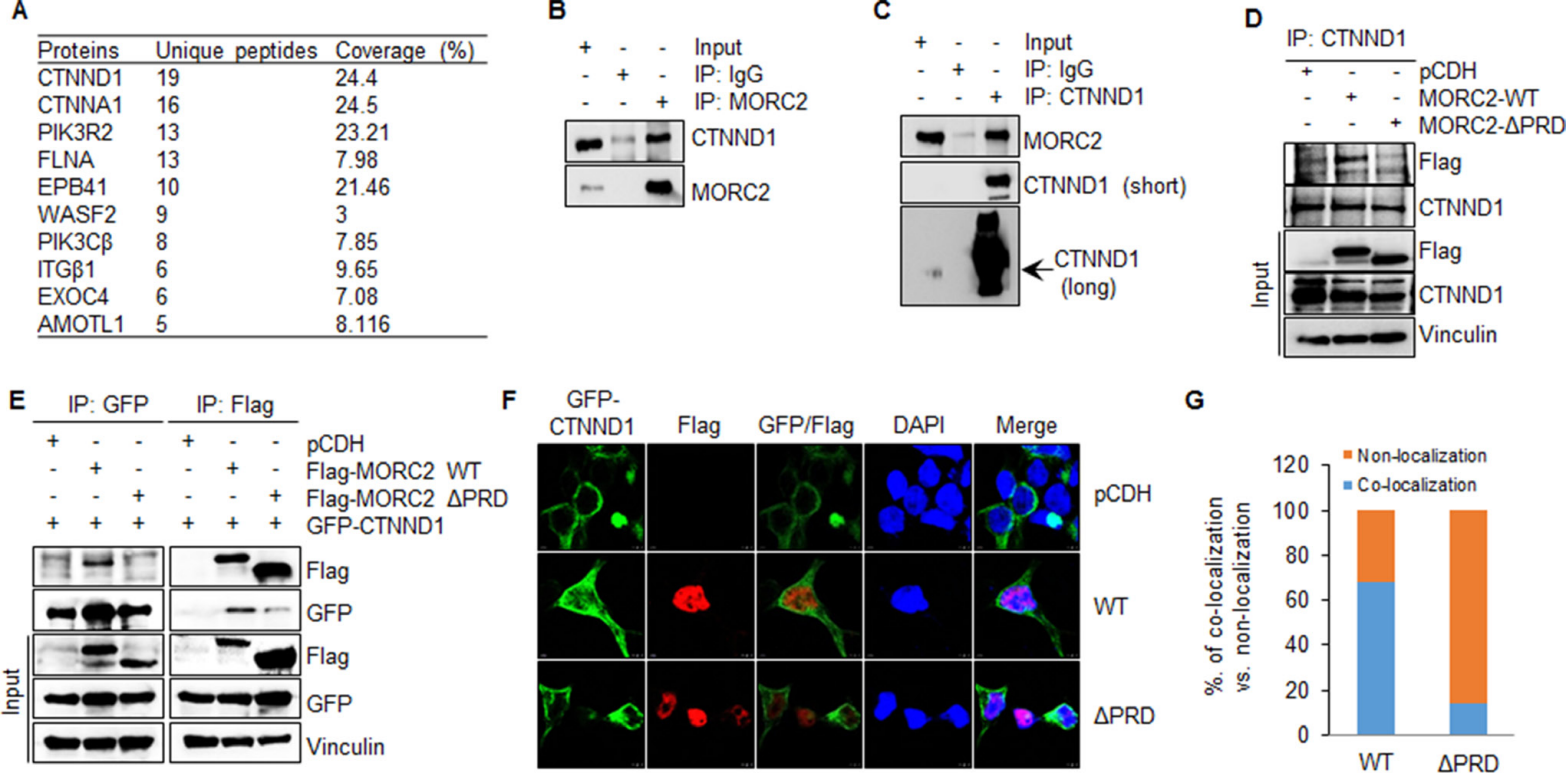
MORC2 interacts with CTNND1 through its PRD domain (**A**) The proteins that specifically interacted with Flag-MORC2 WT were analyzed by matching unique peptide numbers and the percentage of coverage. (**B**, **C**) HEK293T cells were subjected to the sequential IP-Western blot analysis with the indicated antibodies. (**D**) HEK293T cells stably expressing pCDH, Flag-MORC2 WT, and Flag-MORC2 ΔPRD were subjected to IP analysis with an anti-CTNND1 antibody, followed by immunoblotting with the indicated antibodies. (**E**) HEK293T cells were transfected with the indicated expression vectors. After 48 h of transfection, total cellular lysates were subjected to sequential IP-Western blot analysis with the indicated antibodies. (**F**, **G**) HEK293T cells were co-transfected with the plasmids encoding pCDH, Flag-MORC2 WT, and Flag-MORC2 ΔPRD along with GFP-CTNND1, and then immunofluorescence staining was carried out using an anti-Flag antibody (F). Cell nuclei were counterstained with DAPI. The quantitative results of co-localization of GFP-CTNND1 with Flag-MORC2 and Flag-MORC2 ΔPRD are shown in G.

Next, we analyzed these proteins whose functions are involved in protein localization and cytoskeleton regulation by unique peptide numbers and percentage of coverage, and found CTNND1 with the top matching unique peptide and the second highest coverage (Figure 5A). To further confirm these results, total cellular lysates from HEK293T and MCF-7 cells were immunoprecipitated with control IgG or with an anti-MORC2 antibody. Results showed that CTNND1 was detected in the purified MORC2 immune-complex but not control IgG ones (Figure 5B and Supplementary Figure 3A). Reverse IP with an anti-CTNND1 antibody further confirmed these observations (Figure 5C and Supplementary Figure 3B). These results suggest that endogenous MORC2 interacts with endogenous CTNND1 in both HEK293T and MCF-7 cells.

To confirm that the interaction between MORC2 and CTNND1 is dependent on the PRD domain of MORC2, we conducted IP analysis using lysates from HEK293T cells stably expressing pCDH, Flag-MORC2 WT, and Flag-MORC2 ΔPRD, and found that exogenously transfected wild-type MORC2, but not empty vector pCDH or PRD deletion mutant MORC2, could interact with endogenous CTNND1 (Figure 5D). Next, HEK293T cells were transfected with the plasmids encoding pCDH, Flag- MORC2 WT, and Flag-MORC2 ΔPRD together with GFP-CTNND1 expression vector. After 48 h of transfection, whole-cell lysates were immunoprecipitated with an anti-Flag antibody or an anti-GFP antibody. Immunoblotting analysis showed that the exogenously expressed wild-type MORC2, not PRD deletion mutant MORC2, could interact with exogenously expressed CTNND1 (Figure 5E). These results suggest that MORC2 interacts with CTNND1 in a PRD domain-dependent manner.

The functions of CTNND1 largely depend on its subcellular localization [36, 44]. We next examined whether GFP-CTNND1 and Flag-MORC2 could co-localize in HEK293T cells. To do this, HEK293T cells were transiently transfected with GFP-CTNND1 in combination with pCDH, Flag-MORC2 WT, and Flag-MORC2 ΔPRD. The transfection efficiency was about 33% for GFP-CTNND1 (Supplementary Figure 4A) and 15% for Flag-MORC2 WT and Flag-MORC2 ΔPRD (Supplementary Figure 4B). In cells expressing GFP-CTNND1 alone, GFP-CTNND1 was primarily localized to cell membrane (Figure 5F and Supplementary Figure 4C, upper panel). However, coexpression of wild-type MORC2 caused GFP-CTNND1 re-localization from cell membrane to the cytosol or nucleus, where both proteins could co-localize (Figure 5F and Supplementary Figure 4C, middle panel). When Flag-MORC2 ΔPRD was coexpressed with GFP-CTNND1, the co-localization between Flag-MORC2 ΔPRD and GFP-CTNND1 was not consistently observed (Figure 5F and Supplementary Figure 4C, bottom panel). The quantitative results of co-localization of GFP-CTNND1 with Flag-MORC2 and Flag-MORC2 ΔPRD are shown in Figure 5G. These results indicate that exogenously transfected wild-type MORC2, not PRD deletion mutant MORC2, could co-localize with exogenously transfected GFP-CTNND1.

### The migration- and invasion-promoting ability of MORC2 depends on the expression of CTNND1

**Figure 6:**
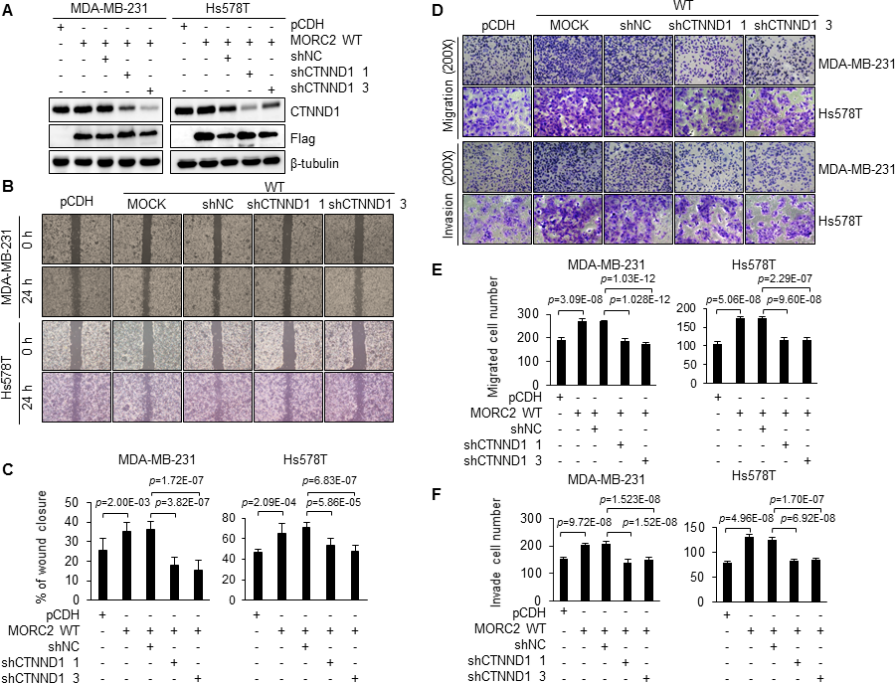
Knockdown of endogenous CTNND1 suppresses MORC2-enhanced cell migration and invasion (**A**) MDA-MB-231 and Hs578T cells stably expressing wild-type MORC2 were infected with two different CTNND1 shRNA expression vectors. The effect of CTNND1 knockdown was verified by immunoblotting with the indicated antibodies. (**B**, **C**) Wound-healing analysis of stable MDA-MB-231 and Hs578T cells expressing the indicated plasmids. Representative images (B) and quantitative results (C) of wound-healing assays are shown. (**D**–**F**) Cell migration and invasion were examined by transwell migration and invasion assay. Representative images (D) and quantitative results of migrated (E) and invaded (F) cell number are shown.

Given that CTNND1 is implicated in the metastatic progression of breast cancer [33, 34, 60] and that MORC2 interacts with CTNND1 (Figure 5), we next assessed the role of CTNND1 in MORC2-enhanced migration and invasion of breast cancer cells. To do this, we knocked down the endogenous CTNND1 in wild-type MORC2 expressing MDA-MB-231 and Hs578T cells using two different short hairpin RNAs (shRNAs). Immunoblotting and qPCR analysis showed that both CTNND1 shRNAs noticeably reduced CTNND1 protein (Figure 6A and Supplementary Figure 5A) and mRNA (Supplementary Figure 5B) levels in both cell lines. Consistent with the above results (Figure 3A–3F), wound healing and transwell migration and invasion assays revealed that overexpression of MORC2 enhanced cell migration and invasion as compared with empty vector control (Figure 6B–6F). In contrast, knockdown of endogenous CTNND1 attenuated MORC2-enhanced cell migration and invasion (Figure 6B–6F). These data suggests that MORC2 promotes migration and invasion of breast cancer cells through, at least in part, a CTNND1 mediated mechanism.

## DISCUSSION

Development of metastatic spread is the leading cause of mortality in patients with breast cancer, thus the elucidation of the molecular determinants of breast cancer cell invasion and metastasis is of crucial importance. The key findings of this work lie in the following. First, it establishes MORC2 as a novel regulator of breast cancer invasion and metastasis. Second, it provides mechanistic insights into the metastasis-promoting activity of MORC2 in breast cancer depending, at least in part, on its PRD domain. Third, it identifies CTNND1 as a novel binding partner of MORC2, which is required for MORC2-mediated breast cancer migration and invasion.

MORC is a poorly characterized, highly conserved nuclear protein family from prokaryotic to eukaryotic cells [6, 61, 62]. The evolutionary contextual and gene neighborhood studies on prokaryotic MORCs predict that their eukaryotic counterparts may be implicated in the regulation of chromatin remodeling through DNA superstructure manipulation [7, 61]. Indeed, our recent study demonstrated that MORC2 is a chromatin remodeling protein in response to DNA damage and is involved in DNA damage repair [7]. Moreover, the role of MORC2 in gene transcription has been documented in gastric cancer cells [8, 10, 11]. Here, we provide *in vitro* and *in vivo* evidence that MORC2 is dispensable for cell proliferation and cell-cycle progression, but promotes breast cancer invasion and metastasis *in vitro* and *in vivo*.

Structurally, MORC2 contains several functional domains, including a conserved GHKL (Gyrase, Hsp90, Histidine kinase, and MutL)-type ATPase domain, a CW-type zinc finger domain (ZF-CW), and a PRD domain [6, 7, 9]. We demonstrated that the ATPase domain is required for the ATP-dependent chromatin remodeling activity of MORC2 following DNA damage [7]. Recent structural and biochemical studies have defined the ZF-CW domain as a histone modification reader module [63]. In contrast, the biological function of the PRD domain in MORC2 has not been characterized. Previous studies have demonstrated that the PRD domain is an important structural module found in diverse tumor suppressor proteins and oncoproteins. For instance, the PRD domain in tumor suppressor protein p53 contributes to the regulation of its protein stability and p53-mediate apoptosis and tumor suppression [54, 55, 64, 65]. The PRD domain in the metastasis-associated in colon cancer protein 1 (MACC1) is required for its oncogenic function in colon cancer growth and metastasis [66]. Similarly, the proline-rich motif is required for the activation and the biological functions of Akt kinase [67]. To address the contribution of the PRD domain to MORC2 functions in breast cancer development and progression, we generated a PRD domain deletion mutant of MORC2 (ΔPRD), and discovered that the PRD domain is dispensable for the stability and subcellular localization of MORC2, but is required for MORC2-mediated migration, invasion, and metastasis.

The PRD domain has been shown to be important for mediating protein-protein interaction [20]. To address the molecular mechanisms by which MORC2 promotes breast cancer invasion and metastasis through its PRD domain, we further identified CTNND1 as a novel MORC2-binding partner by LC-MS/MS and biochemical analyses. CTNND1 is deregulated in the majority of human cancers [25] and could act as either a tumor suppressor or an oncogene depending on its subcellular localization [24]. When expressed in cell membrane, CTNND1 functions as a tumor suppressor by stabilizing E-cadherin [27, 28, 34]. However, loss of E-cadherin during tumor progression leads to the cytoplasmic and nuclear translocation of CTNND1 [33, 35, 44]. Cytosolic CTNND1 drives E-cadherin-deficient cancer cell migration, invasion and metastasis through activation of Rho-family GTPases Rac1 and Cdc42 and inhibition of RhoA activity [33, 40]. Nuclear CTNND1 has been observed in invasive breast cancer [35]. Previous studies have reported that nuclear CTNND1 can interact and functionally antagonize the activity of the transcriptional repressor Kaiso, thus regulating expression of Kaiso target genes including matrix metalloproteinase 7 (MMP7) [48], metastasis-associated gene 2 (MTA2) [68], and Wnt11 (WNT11) [50]. Recently, Wnt11-induced Wnt signaling has been identified as a major paracrine factor driving breast cancer invasion [69]. Our data revealed an essential role for endogenous CTNND1 in MORC2-enhanced migration and invasion of E-cadherin-deficient MDA-MB-231 and Hs578T breast cancer cells (Figure 6). As MORC2 is predominately localized in the nuclear (Figure 1E) [7], we hypothesized that the involvement of MORC2-CTNND1 interaction in breast cancer progression is mainly mediated by nuclear CTNND1. In addition, whether the interaction of MORC2 with CTNND1 could affect the interaction between CTNND1 and Kaiso as well as Kaiso target gene expression is needed to be investigated in the future.

In summary, we provide the evidence for the first time that MORC2 promotes the migratory, invasive and metastatic potential of breast cancer, which depends, at least in part, on its PRD domain. These findings provide significant evidence for the understanding of metastatic mechanisms of breast cancer and exploring new therapeutic strategy for preventing breast cancer metastasis. One limitation of this study is that only two triple-negative breast cancer cell lines (MDA-MB-231 and Hs578T) were used in our study. Thus, whether the metastasis-promoting activity of MORC2 and the interaction between MORC2 and CTNND1 could apply to other breast cancer subtypes remain to be addressed in the future.

## MATERIALS AND METHODS

### Cell culture

Human breast cancer MCF-7, T47D, SK-BR-3, BT474, MDA-MB-231, MDA-MB-436, MDA-MB-468, Hs578T, and HCC1937 cell lines, normal breast epithelial MCF10A and HBL100 cell lines, and human embryonic kidney 293T (HEK293T) cell line were obtained from the Type Culture Collection of the Chinese Academy of Sciences (Shanghai, China). All cell lines were authenticated through monitoring cell vitality, mycoplasma contamination, DNA fingerprinting, and isozymes. MCF10A cells were cultured in DMEM/F12 (Cellgro, Manassas, VA, USA) supplemented with 5% donor horse serum (Gibco, Carlsbad, CA, USA), 10 μg/ml insulin, 20 ng/ml human epidermal growth factor, 0.5 μg/ml hydrocortisone, and 100 ng/ml cholera toxin. Other cell lines were maintained in DMEM or RPMI1640 media (Cellgro) supplemented with 10% fetal bovine serum (FBS) (Gibco). These cell lines were expanded and frozen immediately into numerous aliquots after arrival. The cells revived from the frozen stock were used within 10–15 passages and not exceeding a period of 6 months. All biochemical reagents were purchased from :Sigma-Aldrich (St. Louis, MO, USA) unless otherwise noted.

### Plasmids and transfection

Myc-DDK-tagged human MORC2 and GFP-tagged human CTNND1 expression vectors were purchased from Origene (Rockville, MD, USA). Flag-MORC2 WT and Flag- MORC2 ΔPRD were constructed by PCR amplification (Primers are listed in Supplementary Table 2) and then subcloned into the lentiviral vector pCDH-CMV-MCS-EF1-Puro (System Biosciences, Mountain View, CA, USA). CTNND1 shRNA expression vectors were cloned into pLKO.1-GFP-shRNA expressions vector (Kindly provided by Prof. Xin-Yuan Liu, Institute of Biochemistry and Cell Biology, Chinese Academy of Science, Shanghai, China) using primers listed in Supplementary Table 3. The lentiviral and packaging vectors were transfected into HEK293T packaging cells using Lipofectamine 2000 (Thermo Fisher, Waltham, MA, USA) or Teng-fect (TengYi Biotech, Shanghai, China) transfection reagents. The supernatant containing viruses was collected 48 h after transfection, filtered, and used for infecting target cells in the presence of 8 μg/ml polybrene prior to drug selection with 2 μg/ml puromycin (Cayman Chemical, Ann Arbor, MI, USA) for one week.

### Cell viability, colony-formation assay, and cell cycle analysis

Cells were seeded in 96-well plates (1000 cells per well) in triplicate and cell viability was examined using Cell Counting Kit-8 (CCK-8) kit (Dojindo Laboratories, Kumamoto, Japan). For colony-formation assay, cells were seeded in 6-well plates (1000 cells per well) in triplicate and cultured under normal growth conditions for 2 weeks. Colonies were stained with 0.1% crystal violet and counted. For cell cycle analysis, cells were harvested and fixed in 70% ethanol overnight. After PBS wash, cells were stained with cell cycle staining kit (MultiSciences Biotech, Hangzhou, China), and analyzed on a BD FACSCanto II flow cytometer (BD Bioscience, San Jose, CA, USA).

### Migration, invasion, and lung colonization assays

For wound-healing assays, cells were seeded in 6-well plates. When cells were grown to confluency, the wound was created by 200 μl tips, the floated cells were removed through PBS washing, and the culture medium were replaced by DMEM containing 0.1% FBS. Images were taken at the indicated time points and the wound closure ratios were calculated.

Migration and invasion assays were conducted using 8-μm pore polycarbonate transwell inserts and BioCoat Matrigel Invasion Chambers (Corning, New York, NY, USA), respectively, as described previously [70]. Briefly, cells (transwell migration assays: 2.5 × 10^5^ cells for MDA-MB-231 and 1.0 × 10^5^ cells for Hs578T; transwell invasion assays: 5 × 10^5^ cells for MDA-MB-231 and 2.5 × 10^5^ cells for Hs578T) were plated in the top chamber using growth medium containing 0.5% FBS. Growth medium containing 10% FBS was used as a chemoattractant in the lower chamber. After 24 h, migrated and invaded cells were fixed and stained with 0.1% crystal violet. Cells were counted under an inverted microscope at 200× magnification.

For lung colonization experiments, 2.25 × 10^6^ cells in 300 μl of PBS were injected in the tail vein of 5–6 week-old BALB/c female nude mice (5 mice per group). After 6 weeks of injection, the lungs were excised, fixed in Bouin solution overnight, and surface lung colonies were counted under a Nikon SMZ1500 stereomicroscope (Nikon, Tokyo, Japan). In addition, paraffin-embedded sections were stained by hematoxylin and eosin (HE) to examine the presence of micrometastases. All animal experiments were approved by Institutional Animal Care and Use Committee of Fudan University and animal care was in accordance with institutional guidelines.

### Quantitative real-time PCR (qPCR)

Total RNA was isolated using Trizol reagent (Invitrogen, Carlsbad, CA, USA) and converted to cDNA using PrimeScript RT Master Mix (Takara, Dalian, China). qPCR analyses were performed using FastStart Universal SYBR Green Master (Roche, Shanghai, China). Primer information is described in Supplementary Table 4.

### Antibodies, immunoblotting, immunoprecipitation, and immunofluorescence

The detailed information for primary antibodies used in this study is provided in Supplementary Table 5. Immunoblotting, immunoprecipitation (IP), and indirect immunofluorescence (IF) staining were conducted as described previously [7, 71]. Briefly, protein extracts were prepared using modified RIPA buffer, resolved by SDS-PAGE, and transferred onto PVDF membrane (Millipore, Billerica, MA, USA), followed by antibody detection using enhanced chemiluminescence (Yeasen, Shanghai, China). For IP assay, 1–2 mg of both exogenously and endogenously expressed proteins was incubated with 1–2 μg of the indicated antibodies overnight at 4°C. Protein A/G magnetic beads (Bimake, Houston, TX, USA) were used to pull down the protein-antibody complex. The resulting complexes were washed and subjected to immunoblotting. For indirect IF staining, cells were fixed in 4% paraformaldehyde, permeabilized in 0.1% Triton X-100, and blocked in 10% normal goat serum in PBS. Cells were incubated with primary antibodies, washed three times in PBS, and then incubated with the appropriate secondary antibody conjugated with 555-Alexa (red) or 488-Alexa (green) (Cell Signaling Technology, Danvers, MA, USA), respectively. DNA staining was performed using fluoroshield mounting medium with DAPI (Abcam, Cambridge, MA, USA). Microscopic analyses were performed using a Leica SP5 confocal laser scanning microscopy (Leica Microsystems, Buffalo Grove, IL, USA).

### Proteomic assay

Liquid chromatography tandem mass spectrometry (LC-MS/MS) assay was performed as described previously [72] to analyze MORC2 interacting complex.

### Statistical analysis

All data are presented as the mean ± standard error from at least three independent experiments. The Student's *t*-test was used for assessing the difference between individual groups and *p* ≤ 0.05 was considered statistically significant.

## SUPPLEMENTARY MATERIALS FIGURES AND TABLES





## Abbreviations

CTNND1: catenin delta 1
MORC2: MORC family CW-type zinc finger 2
IF: immunofluorescence
IP: immunoprecipitation
LC-MS/MS: liquid chromatography tandem mass spectrometry
PRD: proline-rich domain.
