# Supplementary material for: Chromatin remodeling protein MORC2 promotes breast cancer invasion and metastasis through a PRD domain-mediated interaction with CTNND1
Source: Oncotarget. 2017 Jun 16;8(58):97941–54. doi: 10.18632/oncotarget.18556 (PMC5716704; doi:10.18632/oncotarget.18556)
Supplement: Supplementary file 1 [file oncotarget-08-97941-s001.pdf]

Chromatin remodeling protein MORC2 promotes breast cancer invasion and metastasis through a PRD domain-mediated interaction with CTNND1

SUPPLEMENTARY MATERIALS

The PRD domain of human P53 protein (61-94 aa)

DEAPRMPEAAPRVAPAPAAPTPAAPAPAPSWPLS

The PRD domain of human MORC2 protein (601-734 aa)

PSTEEPVRRPQRPRSPPLPAVIRNAPSRPPSLPTPRPASQPRKAPVISSTPKLPALAAREEASTSRLL
QPPEAPRKPANTLVKTASRPAPLVQQLSPSLLPNSKSPREVPSPKVIKTPVVKKTESPIKLSPATP

Supplementary Figure 1: Comparison of the PRD domain of human p53 and MORC2 proteins. P indicates proline.

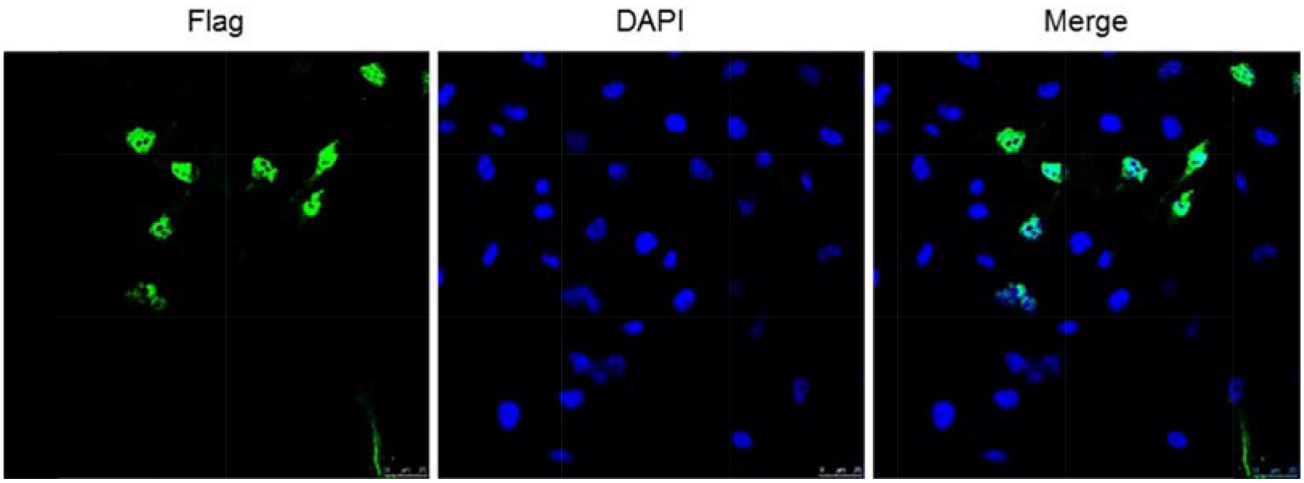

Supplementary Figure 2: Transiently expressed Flag-MORC2 is mainly localized in the nuclear. Cells were infected with lentiviral vector encoding Flag-MORC2 WT for 48 h and then subjected to indirect immunofluorescence staining with an anti-Flag antibody. Cell nuclei were counterstained with DAPI. The infection efficiency of lentiviral vector was about 20% and about 85% transiently expressed Flag-MORC2 was localized in the nuclear.

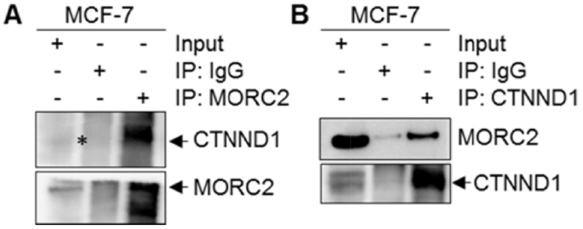

Supplementary Figure 3: Endogenous MORC2 interacts with endogenous CTNND1. Total cellular lysates from MCF-7 cells were subjected to the sequential IP-Western blot analysis with the indicated antibodies.

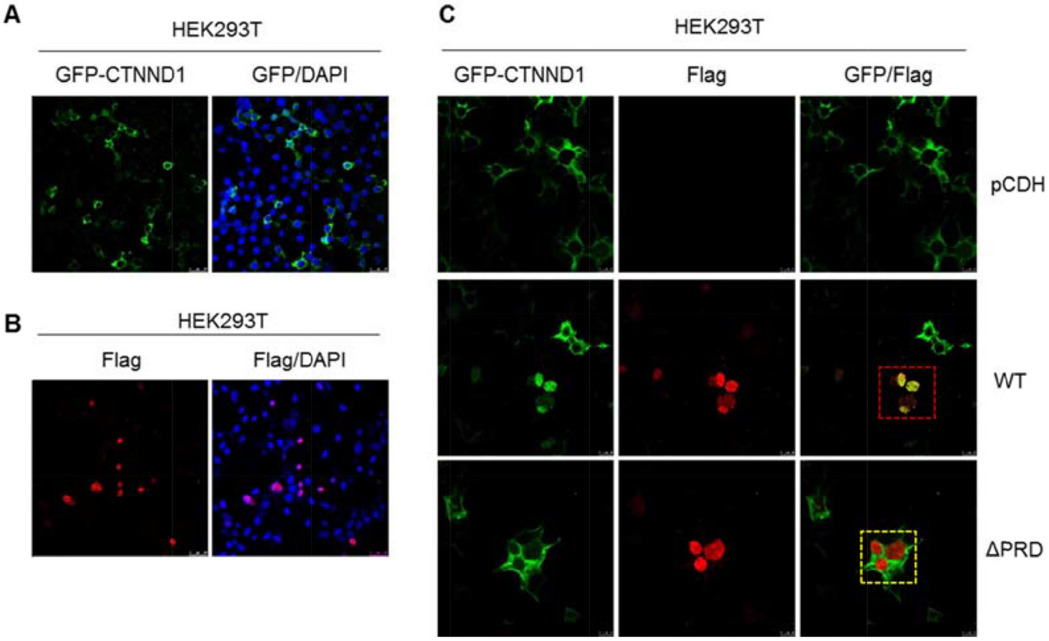

Supplementary Figure 4: Exogenously transfected wild-type MORC2, not PRD deletion mutant MORC2, is mainly co-localized with exogenously transfected GFP-CTNND1. (**A**) HEK293T cells were transfected with GFP-CTNND1. After 48 h of transfection, expression of GFP-CTNND1 was examined under a Laser Scanning Confocal Microscope. Cell nuclei were counterstained with DAPI. The transfection efficiency was about 33% for GFP-CTNND1. (**B**) HEK293T cells were transfected with Flag-MORC2. After 48 h of transfection, immunofluorescence staining was carried out using an anti-Flag antibody. Cell nuclei were counterstained with DAPI. The transfection efficiency was about 15% for Flag-MORC2 WT. (**C**) HEK293T cells were co-transfected with the plasmids encoding pCDH, Flag-MORC2 WT, and Flag-MORC2 ΔPRD along with GFP-CTNND1, and then immunofluorescence staining was carried out using an anti-Flag antibody. Cell nuclei were counterstained with DAPI.

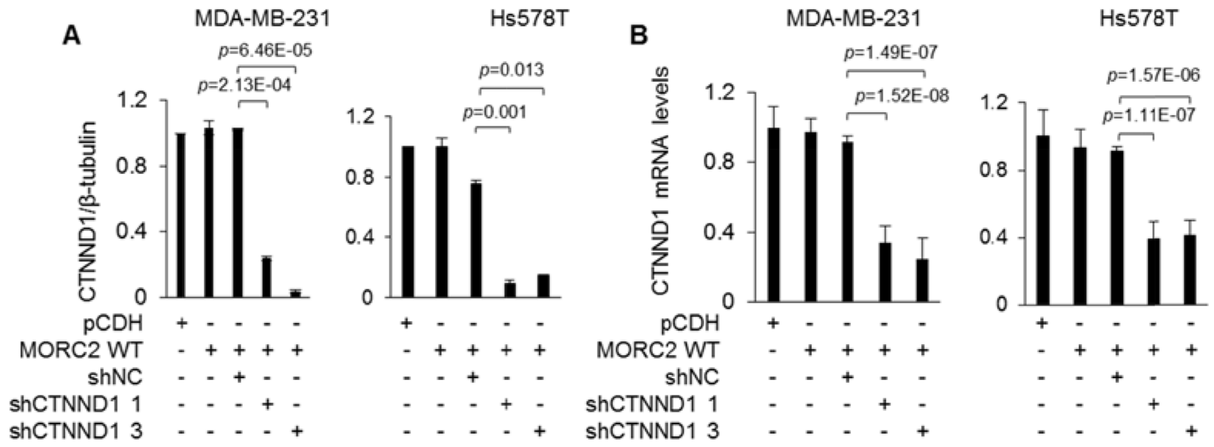

Supplementary Figure 5: Knockdown of endogenous CTNND1 using CTNND1 shRNAs. (**A**) MDA-MB-231 and Hs578T cells stably expressing wild-type MORC2 were infected with two different CTNND1 shRNA (shCTNND1) expression vectors. The effect of CTNND1 knockdown was verified by immunoblotting with the indicated antibodies (Figure 6A). The relative protein expression levels of CTNND1 (CTNND1/β-tubulin) is shown. (**B**) MDA-MB-231 and Hs578T cells stably expressing wild-type MORC2 were infected with two different CTNND1 shRNA expression vectors and then subjected to qPCR analysis of CTNND1 mRNA levels.

Supplementary Table 1: The 536 proteins specifically interacting with wild-type MORC2. See Supplementary Table 1

Supplementary Table 2: Primer sequences for plasmid construction

Name	Sequence
MORC2-1F	ACCTCCATAGAAGATTCTAGAGCCACCATGGCCTTCACCAATTACAGCAGTCTC
MORC2-1R (Flag)	GATCCATTTTTCGAATTCTTACTTGTCGTCATCGTCCTTGTAGTC
MORC2-1030R	ATCGTCCTTGTAGTCGTCCCCTTTGGTGATGAGGTCCT
MORC2-1480F	CCAACCACTATCCAGTGTGATCTG
MORC2-1503R	CAGATCACACTGAGTGGTTGG

Supplementary Table 3: Primer sequences for CTNND1 shRNA construction

Name	Sequence
shCTNND1-1-F	CCGGCTCCCAATGTTGCCAACAATACTCGAGTATTGTTGGCAACATTGGGAGTTTTTG
shCTNND1-1-R	AATTCAAAAACTCCCAATGTTGCCAACAATACTCGAGTATTGTTGGCAACATTGGGAG
shCTNND1-3-F	CCGGCGCCACTATGAAGATGGTTATCTCGAGATAACCATCTTCATAGTGGCGTTTTTG
shCTNND1-3-R	AATTCAAAAACGCCACTATGAAGATGGTTATCTCGAGATAACCATCTTCATAGTGGCG

Supplementary Table 4: Primer sequences for qPCR

Name	Sequence
GAPDH-F	GGAGCGAGATCCCTCCAAAAT
GAPDH-R	GGCTGTTGTCATACTTCTCATGG
CTNND1-F	ATGAAGATGGTTATCCAGGTGGC
CTNND1-R	CTGGGCCTATACCGCTCCT

Supplementary Table 5: The antibodies used in this study

Name	Company	Catalog number
Flag	Sigma	F3165
Vinculin	Sigma	V9131
MORC2	Bethyl	A300-149
CTNND1	Abcam	ab32095
β-tubulin	CST	2146S
GFP	Millipore	06-896
Anti-rabbit IgG (HRP-linked)	CST	7074V
Anti-mouse IgG (HRP-linked)	CST	7076V
Anti-mouse IgG (fluor 488 conjugated)	CST	4408S
Anti-mouse IgG (fluor 555 conjugated)	CST	4409S
Normal rabbit IgG	Santa Cruz	Sc-2027
ERα	CST	8644
HER2	CST	4290p
